# Protein Expression in Tonsillar and Base of Tongue Cancer and in Relation to Human Papillomavirus (HPV) and Clinical Outcome

**DOI:** 10.3390/ijms19040978

**Published:** 2018-03-25

**Authors:** Torbjörn Ramqvist, Anders Näsman, Bo Franzén, Cinzia Bersani, Andrey Alexeyenko, Susanne Becker, Linnea Haeggblom, Aeneas Kolev, Tina Dalianis, Eva Munck-Wikland

**Affiliations:** 1 Department of Oncology-Pathology, Karolinska Institutet, Karolinska University Hospital, 171 76 Stockholm, Sweden; anders.nasman@ki.se (A.N.); bo.franzen@ki.se (B.F.); cinzia.bersani@ki.se (C.B.); susanne.becker@ki.se (S.B.); linnea.haeggblom@ki.se (L.H.); tina.dalianis@ki.se (T.D.); 2 Department of Microbiology, Tumor and Cell Biology (MTC), Karolinska Institutet, 171 77 Stockholm, Sweden; andrej.alekseenko@scilifelab.se; 3 National Bioinformatics Infrastructure Sweden, Science for Life Laboratory, 17121 Solna, Sweden; 4 Department of Clinical Science and Technology (CLINTEC), Karolinska Institutet, Karolinska University Hospital, 171 76 Stockholm, Sweden; aeneas.kolev@sll.se (A.K.); eva.munck-afrosenschold-wikland@sll.se (E.M.-W.)

**Keywords:** tonsillar cancer, base of tongue cancer, oropharyngeal cancer, human papillomavirus, proximity extension assay, clinical outcome, protein expression

## Abstract

Human papillomavirus (HPV) is a major etiological factor for tonsillar and the base of tongue cancer (TSCC/BOTSCC). HPV-positive and HPV-negative TSCC/BOTSCC present major differences in mutations, mRNA expression and clinical outcome. Earlier protein studies on TSCC/BOTSCC have mainly analyzed individual proteins. Here, the aim was to compare a larger set of cancer and immune related proteins in HPV-positive and HPV-negative TSCC/BOTSCC in relation to normal tissue, presence of HPV, and clinical outcome. Fresh frozen tissue from 42 HPV-positive and 17 HPV-negative TSCC/BOTSCC, and corresponding normal samples, were analyzed for expression of 167 proteins using two Olink multiplex immunoassays. Major differences in protein expression between TSCC/BOTSCC and normal tissue were identified, especially in chemo- and cytokines. Moreover, 34 proteins, mainly immunoregulatory proteins and chemokines, were differently expressed in HPV-positive vs HPV-negative TSCC/BOTSCC. Several proteins were potentially related to clinical outcome for HPV-positive or HPV-negative tumors. For HPV-positive tumors, these were mostly related to angiogenesis and hypoxia. Correlation with clinical outcome of one of these, VEGFA, was validated by immunohistochemistry. Differences in immune related proteins between HPV-positive and HPV-negative TSCC/BOTSCC reflect the stronger activity of the immune defense in the former. Angiogenesis related proteins might serve as potential targets for therapy in HPV-positive TSCC/BOTSCC.

## 1. Introduction

The incidence of oropharyngeal squamous cell carcinoma (OPSCC) has increased in many Western countries, due to a rise in incidence of human papillomavirus (HPV) positive tonsillar squamous cell carcinoma (TSCC) and base of tongue squamous cell carcinoma (BOTSCC), the two most commonly HPV-positive OPSCC subsites [1,2,3]. Notably, HPV-positive TSCC/BOTSCC has a much better clinical outcome than the corresponding HPV-negative cancer, with the latter mainly being caused by smoking [4,5,6]. HPV-positive and HPV-negative TSCC/BOTSCC diverge with regard to chromosomal rearrangements, mutations (e.g., p53), RNA and microRNA expression, and DNA methylation [7,8,9,10,11]. Moreover, some proteins differ in expression between HPV-positive and HPV-negative TSCC/BOTSCC and in their relation to clinical outcome (e.g., p16, CD44) [11,12,13]. Most studies on expression at the protein level have been performed on specific proteins by immunohistochemistry (IHC), and thus rather few proteins have been analyzed in total. For this reason, there is a lack of data on differences in protein expression between HPV-positive and HPV-negative TSCC/BOTSCC, as well as between these entities and normal tissue and in relation to clinical outcome. Several studies have demonstrated differences with regard to cellular immune response between these tumor types, but they have mainly targeted differences in cell populations, e.g., CD8+, CD4+ and FoxP3+ T-cells, whereas only few immune related proteins, e.g., PD-1 and APM components, have been specifically evaluated [14,15,16,17,18,19].

Olink multiplex assays are recently developed immunoassays, based on proximity extension assay (PEA) technology, with high sensitivity and reproducibility over a wide range of protein concentrations [20]. Each panel targets 94 cancer and/or immune related proteins, and only requires a small amount of sample. Here, the Olink Oncology II and Immuno-Onc panels were employed to evaluate cancer and immune related proteins in TSCC/BOTSCC in relation to tumor HPV status, normal tissue, and clinical outcome.

## 2. Results

Fifty-nine TSCC/BOTSCC (Table 1) and their corresponding normal samples were analyzed on Olink Immuno-Oncology and Oncology II panels covering 167 proteins. A heat map combined with a dendrogram demonstrated that 58/59 replicates of the tumor samples were positioned as branches that were directly adjoint to each other, so for future analysis an average between the replicate values was used. The final analysis included 155 proteins in 55 tumor and normal samples. More specifically, the analysis of 12 proteins (IL-2, IL-4, IL-5, IL-10, IL-13, IL-21, IL-35, IFN-β, IFN-γ, PPY, SDF-1, SEZ6L) with values that are below the level of detection (LOD, as defined by Olink) in >70% samples and low values in the others, were not included and four tumor/normal sample pairs, where internal and/or detection controls failed, were excluded.

### 2.1. Comparison between Tumor and Normal Samples

A heatmap visualization revealed a clear separation between cancer and normal tissue (Figure S1). HPV-positive and HPV-negative cancers were then compared separately with their corresponding normal samples (HPV-positive tumors with normal samples from patients with HPV-positive tumors, etc.). For HPV-positive tumors vs. normal samples, 111/155 proteins showed significant differences and of these 44 proteins, 38 proteins >4-fold upregulated and 6 >2-fold downregulated in the tumors, are presented in Supplementary Table S1 (Bonferroni-adjusted *p*-values < 0.05, with exception of HK8 for which it was *p* = 0.1). For HPV-negative tumors vs. normal samples 78/155 proteins differed significantly, and 37 proteins (29 >4-fold upregulated and 8 >2-fold downregulated are presented in Supplementary Table S2.

Table 2 presents the 13 proteins with the strongest upregulation in HPV-positive tumors vs. normal samples. The corresponding values for HPV-negative tumors, which are also presented in Table 2, reveal that these proteins were also highly upregulated in those tumors although some (e.g., CCL20 and CXCL11) were apparently less upregulated than in HPV-positive tumors. Most of the proteins in Table 2 were chemokines and cytokines, although e.g., CA9 with >50-fold upregulation is hypoxia related.

### 2.2. Predictive Models for HPV-Positive and HPV-Negative Tumors

Predictive models, being potentially useful for distinguishing between cancer and normal tissues were developed by differential protein expression analysis of HPV-positive and HPV-negative tumors against the normal samples (Figure S2). For HPV positive tumors, the following multiple regression model was obtained; T = −7.3 + 0.62 × WISP1 + 0.44 × CXCL10 + 0.39 × CCL7 − 0.3 × ICOSLG + 0.27 × CA9 − 0.27 × IL-18 + 0.18 × VEGFA − 0.15 × FRα − 0.073 × CD207 + 0.064 × TCL1A + 0.04 × CX3CL1, where the protein names denote the respective expression variables, T is the ‘tumor/normal’ status, and the first term is the intercept of the linear equation. Since the expression values were in the same range after the Olink normalization, the regression coefficients convey proteins’ importance of the specific proteins in this model.

For HPV negative tumors, the model was simpler and shared with the former model for all of the proteins, except CCL4, ANG2, and CCL3: T = −1.6 + 0.47 × CCL7− 0.35 × IL-18 + 0.22 × CCL4 + 0.17 × CA9+ 0.13 × ANG2 + 0.12 × WISP1 + 0.072 × CCL3.

The algorithm that produced these models aimed at a tradeoff between the models’ simplicity and precision. Thus, these protein terms are the least redundant sets sufficient for distinguishing between tumor and normal samples given the absence/presence of the HPV infection. Despite the perfect separation between the tumor and normal samples within this dataset, robustness and reproducibility of the models should be validated using independently collected data in future studies.

### 2.3. Comparison between HPV-Positive and HPV-Negative Tumors

**Figure 1:**
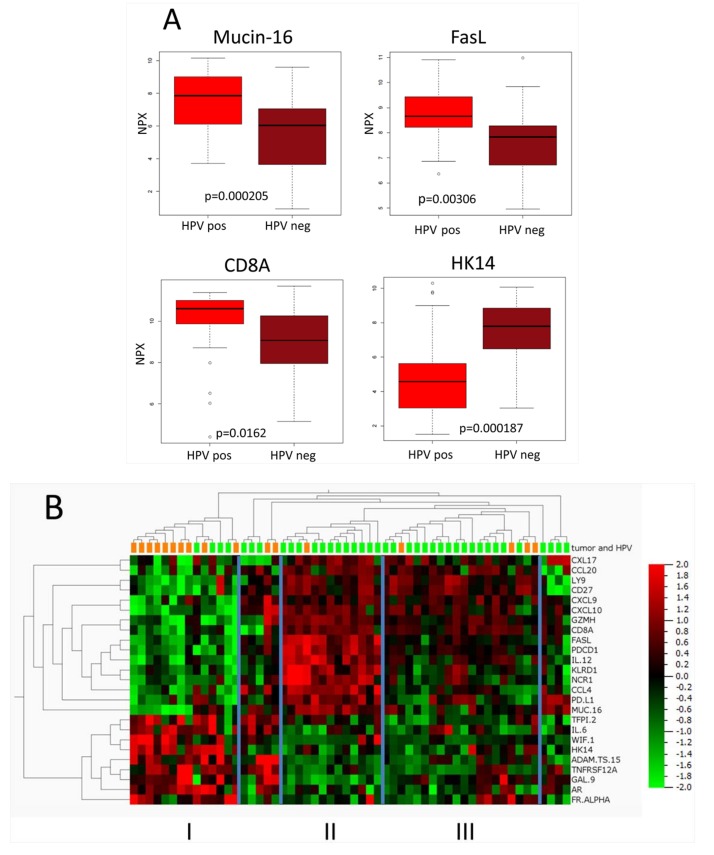
Protein expression in human papillomavirus (HPV) positive and negative TSCC/BOTSCC. (**A**) Boxplots presenting the expression of Mucin-16 (MUC16), Fas ligand (FasL), CD8A, and Human Kallikrein 14 (HK14) in HPV positive and negative TSCC/BOTSCC. NPX-values in 2-log scale. (**B**) Heatmap presenting the expression of 22 proteins with significant differences and >1.5 fold difference between HPV positive and negative TSCC/BOTSCC. Green, HPV positive and orange, HPV negative tumors. Three tentative clusters of samples are marked as I, II, and III.

As noted above, the difference in expression levels between the groups of HPV-positive and HPV-negative tumors was generally less pronounced than between those of tumors vs. normal tissues. Table 3 presents the 34 proteins with significantly different expression in HPV-positive and HPV-negative tumors (unadjusted *p* < 0.05 and FDR < 0.25). Boxplots presenting the expression levels in Mucin-16, FasL, CD8A, and HK14 for HPV-positive and HPV-negative tumors are shown in Figure 1A.

Out of nine proteins with >2-fold higher in expression in HPV-positive tumors, than in HPV-negative tumors, most were surface immunoregulatory proteins that were present on immune or tumor cells (e.g., CD8A, PD-L1, FasL) or chemokines. Notably, however Mucin-16, with a nearly five-fold difference in expression, is a glycoprotein that is present in mucosal surfaces. In contrast, five proteins with >2-fold lower expression in HPV-positive tumors than in HPV-negative tumors were not immune related proteins, e.g., two kallikreins, HK8 and 14, and Wnt inhibitory factor 1 (WIF-1).

In a heatmap, presenting proteins differentially expressed in the HPV-positive as compared with the HPV-negative tumors, most HPV-positive tumors separated into two out of three main clusters (cluster II and III, Figure 1B). These two clusters differed with regard to expression of several membrane molecules related to the immune defense e.g., PD-L1, PD-1, FasL, NCR1, and KLRD1, whereas e.g., CD8A, CD27, CXCL9, and CXCL10 had similar and high expression levels. The third, cluster I, grouped together with the majority of HPV-negative samples, and was characterized by low values of many immune related proteins such as CD8A and the chemokines CXCL9, 10, 17, and CCL20, and by higher values of e.g., Gal9, HK14, ADAMTS15, and IL6.

### 2.4. Protein Expression in Relation to Clinical Outcome

Proteins with formally significant expression differences (unadjusted *p* < 0.05), possibly associated to clinical outcome, are depicted for HPV-positive and HPV-negative tumors in Table 4 and Table 5. Tumor samples from patients, dead of other causes within 4-years, were excluded in this analysis.

**Figure 2:**
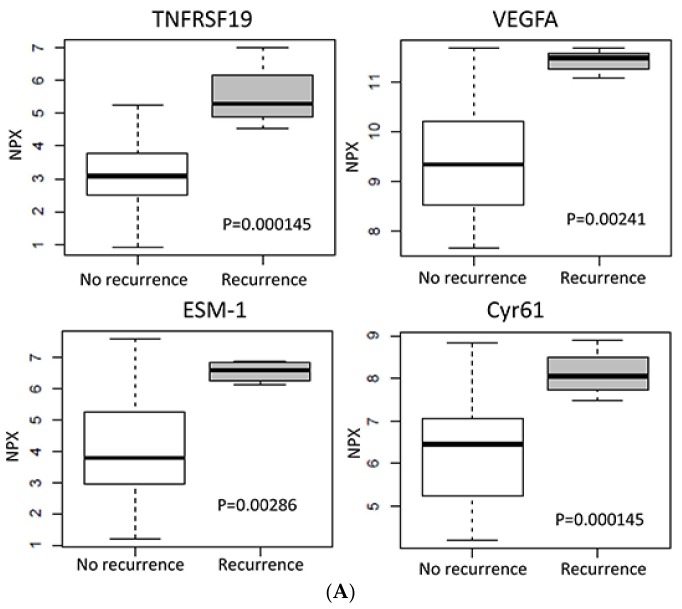

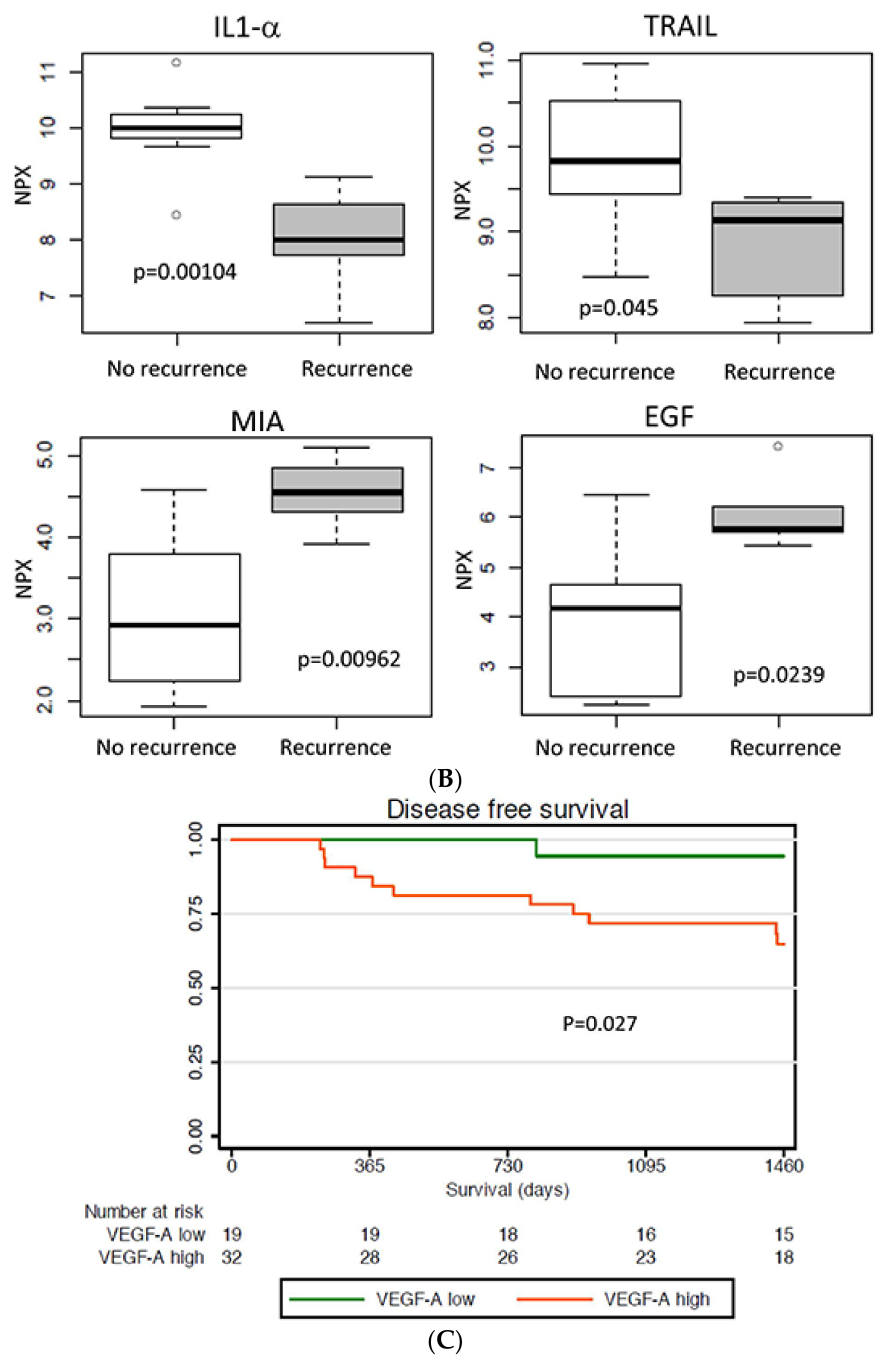
Protein expression in relation to clinical outcome. (**A**) Boxplots presenting the expression of in HPV positive oropharyngeal squamous cell carcinoma (OPSCC) with recurrence vs those with four-year tumor free survival. (**B**) Boxplots presenting the expression of specific proteins in HPV negative OPSCC with recurrence and or death with tumor vs those with four-year tumor free survival. NPX-values in two-log scale. (**C**) Kaplan–Meier curves of disease free survival for 49 patients dichotomized based on tumor VEGFA expression in the validation set. Numbers below the diagram refer to the number of patients at risk at the specific time point.

Notably, despite the noticeable fold change ratios, the *p*-value levels were not always sufficiently low (e.g., only TNFRS19 (Troy) was significant after Bonferroni correction for multiple testing). This could be explained by the relatively small sample sizes and these findings require further validation in an extended cohort. For HPV-positive tumors, 15 proteins were related to recurrence after therapy, all with a higher expression in the primary tumors with later recurrence (Table 4). The 13 proteins with >2-fold higher expression in tumors with recurrence, included proteins that were at least partially related to angiogenesis (DLL1, ESM1, VEGFA, CYR61, and PlGF), and two proteins belonging to the TNF receptors, TNFRSF19 and TNFRSF21. Noteworthy, the IGF receptor IGF1R showed increased expression levels in tumors with recurrence, while no proteins had significantly lower expression. Boxplots for TNFRSF19, VEGFA, ESM-1, and Cyr61 in HPV-positive tumors with/without recurrence are presented in Figure 2A.

In the HPV-negative tumors, 11 proteins were related to recurrence with only three (WFDC2, EGF, and MIA) showing a >2-fold higher expression, while the majority had a lower expression, with IL1α, CXCL11, and TRAIL being the most prominent (Table 5). Expression levels of some proteins that were related to recurrence in HPV negative tumors are presented in boxplots (Figure 2B).

### 2.5. Proteins Related to High CD8A Expression

CD8+ positive tumor infiltrating lymphocytes (CD8+ TILs) have earlier been related to improved prognosis for both HPV-positive and HPV-negative TSCC/BOTSCC, although the latter in general have a lower number of CD8+ TILs [21,22]. For this reason, a specific evaluation of proteins that were related to the expression of CD8A, present in the panel, was of special interest.

This evaluation was performed by correcting for % tumor cells in the sample both for all of the tumor samples taken together and then separately for HPV-positive and HPV-negative tumors. Proteins with *p* < 0.01 for all of these analysis are presented in Table S3. When all tumor samples were evaluated together the correlation of GZMH, ABL1, IL-7, LY9, IL-12, SCAMP3, and CD244 were highly significant (*p* < 1 × 10^−6^). The correlates usually remained significant even after the fraction of tumor cells was accounted for as a covariate, indicating that the analysis was largely unaffected by this factor.

### 2.6. Protein Expression in Relation to Tumor T-Stage

Protein expression was also evaluated in relation to tumor T-stage, by comparing the pooled set of T1/T2 with that of T3/T4, for HPV-positive and HPV-negative tumors separately. Significant differences are presented in Table S4. For HPV-positive tumors, the relation between protein expression and T-stage was minor, with <2-fold ratios of the five proteins with significant differences. For HPV-negative tumors more proteins demonstrated profound differences, with MMP7, CXCL17, and CEACAM5 exhibiting >4-fold higher expression at stage T3/T4 vs. stage T1/T2.

### 2.7. Validation of VEGFA Expression in Relation to Prognosis

To validate the expression of VEGFA in relation to clinical outcome a set of 49 TSCC/BOTSCC biopsies was evaluated for VEGFA expression by IHC. The tumors were then dichotomized based on the VEGFA intensity and analyzed in relation to disease free and progression free survival. Strong VEGFA expression was found to be correlated to poor disease-free survival (DSF), *p* = 0.027, Figure 2C, whereas for progression-free survival (PFS), this tendency failed to reach significance (*p* = 0.107).

## 3. Discussion

In this study, 55 TSCC/BOTSCC biopsies and corresponding normal samples were analyzed for the expression of 155 cancer and immune related proteins, and differences in protein expression between tumor and normal tissue as well as between HPV-positive and HPV-negative TSCC/BOTSCC, were identified. Furthermore, proteins potentially related to clinical outcome, for HPV-positive and HPV-negative tumors, respectively, were identified. One of these proteins, VEGFA, was validated and was shown to be related to disease-free survival for HPV-positive TSCC/BOTSCC.

The proteins included in the Olink panels utilized in the present study, had been selected as directly or indirectly cancer related and involved in processes such as angiogenesis, cell-cell signaling, cell-cycle control, tumor-immunity, chemotaxis, apoptosis, and cell killing (www.olink.com/products). Therefore, it was not surprising that many differences in protein expression between tumor and normal tissue were found. That fewer (78/155) proteins differed in expression between HPV-negative tumors as compared to HPV-positive tumors (111/155) vs. their corresponding normal samples was likely a combined effect of a smaller cohort (i.e., lower statistical power) and/or a less active immune defense in the former.

The most upregulated proteins in the tumor tissue were mainly chemokines and cytokines, indicating an increased activity of the immune response, as especially shown for chemokines CXCL9, 10, 11, 13, and CCL3, 4 and 20, and cytokines IL-6 and 8. A recent analysis of 20 different cytokines and chemokines (some also included here) in culture supernatants harvested from HNSCC tumor tissue-derived cell suspensions, showed, in accordance with the present study, high tumor related levels of CXCL9, CXCL10, and CCL20 and decreased levels of CCL5 [23]. In parallel to this study, higher expression of CXCL9 and CXCL10 was noted in HPV-positive tumors than in HPV-negative tumors. Furthermore, in a study on melanoma a 12-chemokine signature that was related to immune infiltrates included high expression of CXCL9, 10, 11, 13, and CCL 3 and 4, similarly to TSCC/BOTSCC in the present study [24].

Proteins with a distinct lower expression in HPV-positive TSCC/BOTSCC vs. normal samples included kallikreins KLK13, HK8, and HK11. Kallikreins have mostly been investigated as serum markers in prostate cancer, but also in some other cancer types e.g., lung cancer where HK8 has been found to be related to favorable outcome and shown to suppress tumor invasiveness in lung cancer, while little is known about their expression in HNSCC and TSCC/BOTSCC [25].

Proteins with higher expression in HPV-positive vs. HPV-negative TSCC/BOTSCC were mainly immune related. Several were surface proteins expressed on immune cells, e.g., CD8A, and PD-1, or on cancer cells and affecting immune activity, e.g., PD-L1, FASL, and notably PD-1 and its ligand PD-L1 were similarly upregulated. A higher CD8A expression was expected, since increased numbers of CD8+, FoxP3+, and CD4+ TILs in HPV-positive vs. HPV-negative OPSCC have been noted earlier, where in particular a higher number of CD8+TILs is linked to a better clinical outcome, irrespective of the HPV status of the tumor [19,21,22].

PD-1 and PD-L1 exhibited a higher expression in HPV-positive when compared to HPV-negative tumors in this study. Interaction of PD-1, expressed on the surface of activated T-cells, B-cells, and macrophages, and PD-L1, expressed on immune and cancer cells can suppress the activity of CD8+ T-cell mediated immune response, and have therefore received attention for immunotherapy [26,27]. Recent studies investigating PD-L1 and PD-1 expression in OPSCC gave partly contradictory results, with two studies showing higher PD-L1 mRNA levels in HPV-positive cancer, while the third study reported the opposite result [19,23,28]. Notably, since PD-L1 is expressed on both cancer and tumor infiltrating immune cells, this study, similarly to global mRNA analysis, does not differentiate the expression between these cell types.

In parallel with the higher immune infiltration in HPV-positive than in HPV-negative tumors, some cytokines and chemokines e.g., IL12, CCL4, CCL20, CXCL10, CXCL17 had higher expression in the former, with IL12, notably being an important regulator of T-cell and NK-cells cytotoxicity, and its receptor, IL12R1-β1, was also upregulated in the HPV-positive tumors.

Finally, Mucin-16 (also known as MUC-16), a transmembrane protein that functions as a barrier to bacterial infections, protects cancer cells from being killed by immune cells and contains the CA125 peptide [29], demonstrated a nearly 5-fold difference between HPV-positive and HPV-negative tumors. Mucin-16/CA125 has especially been studied in ovarian cancer where it is used both as a diagnostic and predictive marker and to evaluate the response to therapy in this tumor type [29]. The reason behind the increased expression in HPV-positive vs. HPV-negative TSCC/BOTSCC is yet unclear and needs further investigation.

Few other studies have analyzed a large number of proteins simultaneously in OPSCC in relation to tumor HPV status and/or clinical outcome. Proteomic profiling of OPSCC by mass spectrometry disclosed e.g., enrichment of E2F1 and E2F4 in HPV-positive OPSCC, while reverse-phase protein array profiling revealed differences in e.g., P13K/AKT/mTOR and receptor kinase pathways [30,31].

Here, most HPV-positive tumors separated into clusters, mainly due to differences in the expression of immune related proteins on the cell surface of tumor infiltrating immune cells, e.g., PD-1, FasL, NCR1, and KLRD1 or soluble cytokines/chemokines e.g., IL12 and CCL4 (Figure 1B). High expression of FasL, PD-1, and KLRD1 indicates a high infiltration of several parts of the immune defense with PD-1, mainly expressed on T-, B-cells and macrophages, and NCR1 and KLRD1 mainly expressed on NK-cells [32,33]. Notably, CD8A expression did not differ between cluster II and III indicating no major differences in numbers of CD8+ T-cells between these clusters, in contrast to that observed between HPV-positive and HPV-negative tumors. Similarly, several of the chemokines differing in the expression between HPV-positive and HPV-negative tumors do not differ between these clusters either, e.g., CXCL9 and 10.

Global mRNA expression between HPV-positive and HPV-negative OPSCC has also shown differences in proteins not included in the present study e.g., CDKN2A, NF-KB, and STAT3, making comparisons difficult [34,35,36]. Notably, in one study HPV-positive OPSCC was split into two subtypes, a Classical subtype (CL)-HPV and an Inflamed/mesenchymal subtype (IMS)-HPV, where the latter was characterized by higher expression of e.g., immune response genes, such as CD8A, ICOS, LAG3, and HLA-DRA, related to CD8 T-cell infiltration [36]. Here, out of these, only CD8A was analyzed and it did not present differences between the two major clusters although HPV-positive tumors in a third cluster (I), dominated by HPV-negative tumors, presented a low amount of CD8A expression.

In this study, some of the proteins with the most pronounced potential correlation to clinical outcome in HPV-positive TSCC/BOTSCC were at least partly related to angiogenesis e.g., DLL1, ESM1 (endocan), VEGFA, CYR61, and PlGF. Higher VEGFA expression in the tumor cells was confirmed by IHC to be associated with poorer DSF. High VEGFA expression, especially in combination with high EGFR expression has earlier been linked to local recurrence in TSCC in one study, and likewise in oral and laryngeal cancer, but not in a third study, including HPV-positive and HPV-negative OPSCC [37,38,39,40]. Notably, VEGFA induces the expression of ESM1, which is a mediator of the angiogenic effect of VEGFA [41]. Moreover, ESM1 has been related to shorter survival in e.g., breast, liver, and nasopharyngeal cancer, but has not been investigated in OPSCC [42,43].

Angiogenesis is closely linked to hypoxia, and VEGFA together with CA9, are also markers for hypoxia, both being regulated by HIF1α and are involved in the induction of members of the Notch-pathway where DLL1 functions as a ligand for Notch1 [44]. Also, PlGF, which is a ligand for VEGFA, is induced by hypoxia [45].

Noteworthy, the IGF receptor IGF1R also demonstrated a higher expression in tumors that recurred. IGF1R is potentially targetable for therapy and can inhibit the effect of anti-EGFR therapy by acting on the same downstream pathway [46,47]. Since current TSCC and BOTSCC therapy often includes EGFR inhibitors, there is a potential need to combine such treatment with anti-IGF1R therapy. IGF1R has also previously been linked to poor prognosis in HPV-negative OPSCC [48].

There is a need for new targets for therapy of TSCC and BOTSCC irrespective of HPV-status. Recently, earlier treatment that entailed conventional radiotherapy and/or surgery has been intensified by increased radiotherapy, chemoradiotherapy, and/or EGFR-inhibitors. Intensified treatment leaves the patients with more side effects, but has not improved survival for patients with HPV-positive cancer in Stockholm, Sweden [49]. Treatment for recurrent TSCC and BOTSCC is a real challenge and the results are poor. Proteins related to recurrence in the present study, such as e.g., IGF1R and TNFRSF19/Troy, may possibly be utilized as such targets.

Protein expression was also analyzed in relation to tumor T-stage and major differences related to T-stage were found for some proteins in HPV-negative tumors, but not for HPV-positive tumors. Although T-stage has been shown to be related to clinical outcome [49] the proteins with the strongest relation to T-stage for HPV-negative tumors, MMP7, CSCL17, and CEACAM5, were not found to be related to survival. There are limitations in the present study. Only 59 tumors were included, and when HPV-positive and especially HPV-negative tumors were analyzed separately or compared, random correlations might have been obtained. In addition, both the treatment and T-stage of the tumors included in the cohort were heterogeneous, as presented in Table 1. For proteins related to clinical outcome these issues may have had an effect on the results obtained, especially given the few events. Thus, the result presented here must be interpreted with caution.

In summary, when comparing protein expression in TSCC/BOTSCC with normal tissue, the most prominent differences were found for chemo-and cytokines. Between HPV-positive vs. HPV-negative tumors, most of the differences were detected in the immune related proteins as well as cyto-and chemokines. Some proteins were tentatively related to clinical outcome. For HPV-positive tumors, such proteins were mostly related to hypoxia and angiogenesis, and some may be potentially targetable and need to be further evaluated.

## 4. Material and Methods

### 4.1. Patients and Tumor Biopsies

Fifty-nine study specific pre-treatment tumor biopsies of TSCC (ICD-10 code C09.0-9) and BOTSCC (ICD-10 code C01.9), and adjacent normal tissue, from patients treated 2002–2011 at the Karolinska University Hospital were snap frozen and stored at −70 °C until the cutting of the samples. Corresponding diagnostic formalin fixed paraffin embedded (FFPE) pre-treatment biopsies from the tumors had previously been analyzed for HPV DNA and p16INK4A (p16) overexpression, where being positive for both was defined as HPV-positive status [11]. Patient and tumor characteristics are presented in Table 1, where the AJCC/UICC 7th ed is used for staging. Validation of VEGFA expression was performed on FFPE TSCC/BOTSCC pretreatment biopsies from patients treated 2000–2010 at the Karolinska University Hospital (Table 1). The study was performed according to permissions 02-009 (4 February 2002) and 2009/1278-31/4 (2 September 2009) from the Regional Ethics Committee, Karolinska Institutet. Informed consent was obtained from all of the patients.

### 4.2. Sample Preparation

Biopsies were cut frozen, embedded in optimal cutting temperature compound (OCT), and six cuts/biopsy were made, 1 × 5 μm, 4 × 20 μm and 1 × 5 μm. The first and last were used for haematoxylin-eosin-staining and evaluation of tumor content by an experienced pathologist. All of the tumor samples included had at least 40% tumor cells and 40/59 had >70% tumor cells (taking tumor infiltrating immune cells into account). For tumors, the remaining cuts (20 μm each) were paired 2 and 2, with each pair dissolved and analyzed separately, to obtain two replicates. For normal tissues, the remaining cuts were all pooled and dissolved together. Samples were then dissolved in different amounts of RIPA-buffer (50 mM Tris-HCl pH 7.4, 150 mM NaCl, 1 mM EDTA, 1 % Triton X-100, 0.1% Sodium deoxycholate with protease inhibitors) in relation to the area of the cuts to obtain a standardized protein concentration. Dissolved samples were kept frozen at −70 °C until analysis on the Olink platform.

### 4.3. Analysis on Olink Panels

Sample aliquots were analyzed with two Olink multiplex immunoassays, Immuno-Oncology and Oncology II, (Olink Bioscience, Uppsala, Sweden) at the Clinical Biomarkers facility, Science for Life Laboratory, Uppsala University, with each panel evaluating the concentration of 92 different proteins. In total, 167 unique proteins were analyzed, since 17 proteins overlapped between the two assays (Supplementary Table S5). The 17 common proteins served as a validation of the results between the assays. Concentrations of each protein were reported as normalized protein concentration (NPX) in a 2-log scale, and limit of detection (LOD) was defined as three standard deviations above background [20]. The assays also included two internal controls and a detection control. Quality control and data pre-processing (including normalization) of PEA data was made according to the manufacturer’s recommended procedures. NPX-values for proteins and samples included in the evaluations are presented in Supplementary Table S6.

### 4.4. Evaluation of Data from Olink Panels

In order to enable as much tools of parametric statistics, such as Pearson linear correlation, *t*-test, regression modeling, the expression profiles were rendered normally distributed by taking log2 of the NPX values. The multiple regression model (Figure S2) was created using R package glmnet (available from http://web.stanford.edu/~hastie/glmnet/glmnet_alpha.html) under alpha = 1 and other parameters set to their defaults. *p*-values from *t*-tests were adjusted to report either family-wise error rates (Bonferroni correction) or the false discovery rate [50].

### 4.5. Heatmaps

Heatmap in Figure 1B was generated using Qlucore Omics Explorer 3.2 (Qlucore, Lund, Sweden). Heatmap in Figure S1 was created with R package heatmaply (https://cran.r-project.org/package=heatmaply).

### 4.6. Box Plots of Protein Expression

Using the default settings in R function boxplot, the boxes contain data points within the 25–75th percentile intervals (i.e., in the 2nd and 3rd quartiles). The maximal whisker length, MWL, is defined as 1.5 times the box length. Whiskers can extend to either the MWL or to the maximal available data point when the latter is below MWL. Separate markers thus correspond to data points that extend off the box by more than the MWL value.

### 4.7. Immunohistochemistry for VEGFA

Evaluation of VEGFA expression was performed only in samples that were both HPV DNA and p16 positive and the validation set is presented in Table 1. More specifically, 4 µm FFPE sections were de-paraffinized in xylene and rehydrated with ethanol. Blocking for endogenous peroxidase was performed using 1% bovine serum albumin in TBS. The sections were then incubated with VEGF antibody C1 (Santa Cruz Biotechnology, Heidelberg, Germany), 1:100 overnight at +4 °C, washed three times in TBS, incubated 30 min with Biotinylated horse-anti-mouse antibody diluted 1:200 in TBS with 0.2% Triton-X, and washed three times in TBS. After incubation with ABC-peroxidase (Vector Laboratories, Burlington, CA, USA) for 30 min at RT and three washes in TBS the staining was developed with DAB-kit (Vector Laboratories) for 3 min. Counterstaining was done with haematoxylin and the sections were dehydrated using increasing concentrations of ethanol and xylene before mounting.

Staining was evaluated by two researchers, including one pathologist, blinded for all other information about the samples. For cases where the evaluation differed, a consensus was reached. Staining intensity on tumor cells was scored as 0, absent, 1, weak, 2, moderate, and 3, strong. The percentage of stained tumor cells was assessed to the nearest 10%. Cases where the staining was not possible to be evaluated adequately were excluded.

### 4.8. Evaluation of Survival

For analysis of survival in relation to protein expression in the PEA-assay, tumors were analyzed separately for HPV-positive and HPV-negative tumors and tumors from patients being tumor-free and alive after four-years were compared with tumors from patients with recurrence within and/or dead with tumor within four-years. There were five recurrences/death with tumor among patients with HPV-negative tumors and four recurrences among patients with HPV-positive tumors.

For analysis of survival in relation to VEGF expression by IHC, survival was measured in days from diagnosis until an event occurred, or until four years after diagnosis when the patients were censored. Disease recurrence (TSCC or BOTSCC) was used as event for calculation of disease free survival (DFS) and disease recurrence or death with tumor was used when calculating progression-free survival (PFS). Kaplan-Meier estimator was used for DFS and PFS, and the differences in the survival of patients were tested using the logrank test. The Cox proportional hazards model was used for the calculation of the adjusted and unadjusted hazard ratios (HRs). Calculations and analyses were performed using SPSS Statistics, Version 21.0 (IBM Corp., Armonk, NY, USA).

## Figures and Tables

**Table 1:** Patient and tonsillar squamous cell carcinoma (TSCC)/base of tongue squamous cell carcinoma (BOTSCC) characteristics.

Patient and Tumor Characteristics			HPV+TSCC/BOTSCC (*n* = 42)		HPV-TSCC/BOTSCC (*n* = 17)		All TSCC/BOTSCC (*n* = 59)		Validation set (*n* = 49)	
			*n*	%	*n*	%	*n*	%	*n*	%
Age	Mean (years)		61.7		60.6		61.4		62.1	
	Median (years)		61		64		61		61	
	Range (years)		46–84		32–83		32–84		42–84	
Diagnose	malignant neoplasm of the base of tongue (C01.9)		9	21%	9	53%	18	31%	13	22%
	malignant neoplasm of the tonsil (C09.0-9)		33	79%	8	47%	41	69%	36	61%
Sex	female		8	19%	3	18%	11	19%	13	22%
	male		34	81%	14	82%	48	81%	36	61%
Tumour differentiation	poorly		21	50%	9	53%	30	51%	29	49%
	moderatley		17	40%	8	47%	25	42%	18	31%
	well		2	5%	0	0%	2	3%	1	2%
	undefined		2	5%	0	0%	2	3%	1	2%
Tumour size	T1		6	14%	3	18%	9	15%	8	14%
	T2		16	38%	2	12%	18	31%	20	34%
	T3		11	26%	5	29%	16	27%	10	17%
	T4		9	21%	7	41%	16	27%	11	19%
Nodal disease	N0		5	12%	6	35%	11	19%	5	8%
	N1		12	29%	1	6%	13	22%	11	19%
	N2a		5	12%	3	18%	8	14%	5	8%
	N2b		15	36%	3	18%	18	31%	21	36%
	N2c		5	12%	2	12%	7	12%	7	12%
	N3		0	0%	1	6%	1	2%	0	0%
	NX		0	0%	1	6%	1	2%	0	0%
Distant metastasis	M0		42	100%	16	94%	58	98%	48	81%
	M1		0	0%	0	0%	0	0%	0	0%
	MX		0	0%	1	6%	1	2%	1	2%
Tumour Stage	I		0	0%	3	18%	3	5%	0	0%
	II		4	10%	1	6%	5	8%	3	5%
	III		11	26%	2	12%	13	22%	10	17%
	IVa		27	64%	9	53%	36	61%	34	58%
	IVb		0	0%	1	6%	1	2%	0	0%
	IVc		0	0%	0	0%	0	0%	1	2%
	Unknown		0	0%	1	6%	1	2%	0	0%
Treatment	Induction chemotherapy and radiation	conventional	4	10%	3	18%	7	12%	8	14%
		accelerated	11	26%	9	53%	20	34%	14	24%
	Radiation	conventional	20	48%	4	24%	24	41%	21	36%
		accelerated	7	17%	1	6%	8	14%	6	10%
Brachytherapy boost	Not administered		34	81%	12	71%	46	78%	38	64%
	Administered		8	19%	7	41%	15	25%	11	19%
Concomittant Cetuximab	Not administered		35	83%	13	76%	48	81%	42	71%
	Administered		7	17%	4	24%	11	19%	7	12%
Smoking	Never		15	36%	2	12%	17	29%	16	27%
	Former (>15 years ago)		11	26%	1	6%	12	20%	11	19%
	Former (<15 years ago)		7	17%	3	18%	10	17%	11	19%
	Current upon diagnosis		9	21%	11	65%	20	34%	11	19%

**Table 2:** Proteins with the most prominent differences between TSCC/BOTSCC and normal samples.

Protein	HPV Positive TSCC/BOTSCC vs. Normal		HPV Negative TSCC/BOTSCC vs. Normal	
	Ratio	*p*-Value	Ratio	*p*-Value
CA9	55.72	1.10 × 10^−21^	50.56	1.80 × 10^−8^
CXCL13	37.27	3.70 × 10^−14^	21.26	1.40 × 10^−5^
CXCL10	36.25	1.40 × 10^−19^	15.89	1.50 × 10^−6^
CCL4	35.75	9.30 × 10^−24^	20.25	1.80 × 10^−9^
MMP-12	35.51	4.60 × 10^−18^	33.36	3.90 × 10^−9^
CCL3	34.78	1.30 × 10^−20^	38.85	3.70 × 10^−10^
CCL20	25.81	5.40 × 10^−13^	11.31	0.00011
CXCL11	22.32	3.90 × 10^−18^	11.39	6.30 × 10^−6^
IL-6	20.97	3.40 × 10^−15^	33.59	3.40 × 10^−8^
CXCL9	19.43	2.50 × 10^−18^	9.78	7.70 × 10^−6^
IL-8	19.16	5.90 × 10^−15^	24.42	1.20 × 10^−7^
TNFRSF9	18.25	1.50 × 10^−15^	11	3.80 × 10^−6^
WISP-1	16.34	2.30 × 10^−23^	13.64	2.10 × 10^−9^

**Table 3:** Protein with significant differences in expression between HPV positive and negative TSCC and BOTSCC.

Protein	Ratio HPV-Positive/HPV-Negative (Linear)	*p*-Value	False Discovery Rate (FDR)
MUC-16	4.89	0.0016	0.062
CXCL17	2.73	0.025	0.162
CCL20	2.51	0.0046	0.101
CD8A	2.23	0.024	0.162
FASL	2.23	0.0065	0.101
CCL4	2.20	0.00093	0.057
CXCL10	2.19	0.037	0.186
PD-L1	2.13	0.0024	0.062
IL-12	2.10	0.025	0.162
LY9	1.88	0.026	0.162
CD27	1.83	0.025	0.162
PDCD1	1.62	0.031	0.173
KLRD1	1.61	0.018	0.148
DKN1A	1.53	0.031	0.173
GZMH	1.52	0.046	0.209
NCR1	1.51	0.016	0.148
IL12RB1	1.45	0.015	0.148
CPE	1.45	0.047	0.209
IL-7	1.37	0.016	0.148
VEGFC	0.89	0.046	0.209
ITGAV	0.78	0.017	0.148
HO-1	0.74	0.0096	0.120
GZMB	0.74	0.01	0.120
FURIN	0.73	0.035	0.186
TXLNA	0.72	0.027	0.162
GPNMB	0.70	0.0054	0.101
TNFRSF12A	0.66	0.046	0.209
GAL-9	0.59	0.0011	0.057
ADAMTS15	0.59	0.009	0.120
HK8	0.45	0.037	0.186
TFPI2	0.35	0.0061	0.101
AR	0.34	0.0024	0.062
WIF-1	0.24	0.018	0.148
HK14	0.16	0.00032	0.050

**Table 4:** Proteins related to recurrence in HPV positive TSCC and BOTSCC.

Protein	Ratio * (Linear)	*p*-Value
DLL1	8.38	0.000422
ESM-1	5.76	0.00286
TNFRSF19	5.41	0.000145
VEGFA	4.01	0.00241
CYR61	3.64	0.0044
CCL7	3.23	0.0402
PLGF	2.89	0.0127
MIC-A/B	2.67	0.0452
ANG-2	2.33	0.00218
TNFRSF21	2.20	0.0411
IGF1R	1.78	0.00847
CSF-1	1.74	0.0361
GPNMB	1.62	0.0347
ICOSLG	1.41	0.0441
IFN-GAMMA-R1	1.40	0.037

* Ratio between tumors with recurrence vs tumors from patients with 4-year disease free survival.

**Table 5:** Proteins related to recurrence in HPV negative TSCC and BOTSCC.

Protein	Ratio * (Linear)	*p*-Value
WFDC2	4.11	0.000339
EGF	4.03	0.0239
MIA	2.84	0.00962
VEGFR-2	0.76	0.0275
EPHA2	0.68	0.0167
DKN1A	0.58	0.0382
ANG2	0.52	0.00641
TNFRSF4	0.51	0.0449
TRAIL	0.50	0.045
CXCL11	0.26	0.0462
IL-1-ALPHA	0.26	0.00104

* Ratio between tumors with recurrence vs tumors from patients with 4-year disease free survival.

## Supplementary Materials

Supplementary materials can be found at http://www.mdpi.com/1422-0067/19/4/978/s1.

## References

[1] Haeggblom L., Ramqvist T., Tommasino M., Dalianis T., Nasman A. (2017). Time to change perspectives on hpv in oropharyngeal cancer. A systematic review of hpv prevalence per oropharyngeal sub-site the last 3 years. Papillomavirus Res..

[2] Marklund L., Näsman A., Ramqvist T., Dalianis T., Munck-Wikland E., Hammarstedt L. (2012). Prevalence of human papillomavirus and survival in oropharyngeal cancer other than tonsil or base of tongue cancer. Cancer Med..

[3] Ramqvist T., Dalianis T. (2010). Oropharyngeal cancer epidemic and human papillomavirus. Emerg. Infect. Dis..

[4] Dahlstrand H., Dahlgren L., Lindquist D., Munck-Wikland E., Dalianis T. (2004). Presence of human papillomavirus in tonsillar cancer is a favourable prognostic factor for clinical outcome. Anticancer Res..

[5] Lindquist D., Romanitan M., Hammarstedt L., Nasman A., Dahlstrand H., Lindholm J., Onelov L., Ramqvist T., Ye W., Munck-Wikland E. (2007). Human papillomavirus is a favourable prognostic factor in tonsillar cancer and its oncogenic role is supported by the expression of e6 and e7. Mol. Oncol..

[6] Gillison M.L., D’Souza G., Westra W., Sugar E., Xiao W., Begum S., Viscidi R. (2008). Distinct risk factor profiles for human papillomavirus type 16-positive and human papillomavirus type 16-negative head and neck cancers. J. Natl. Cancer Inst..

[7] Slebos R.J., Yi Y., Ely K., Carter J., Evjen A., Zhang X., Shyr Y., Murphy B.M., Cmelak A.J., Burkey B.B. (2006). Gene expression differences associated with human papillomavirus status in head and neck squamous cell carcinoma. Clin. Cancer Res..

[8] Pyeon D., Newton M.A., Lambert P.F., den Boon J.A., Sengupta S., Marsit C.J., Woodworth C.D., Connor J.P., Haugen T.H., Smith E.M. (2007). Fundamental differences in cell cycle deregulation in human papillomavirus-positive and human papillomavirus-negative head/neck and cervical cancers. Cancer Res..

[9] Dahlgren L., Mellin H., Wangsa D., Heselmeyer-Haddad K., Bjornestal L., Lindholm J., Munck-Wikland E., Auer G., Ried T., Dalianis T. (2003). Comparative genomic hybridization analysis of tonsillar cancer reveals a different pattern of genomic imbalances in human papillomavirus-positive and -negative tumors. Int. J. Cancer.

[10] Lajer C.B., Garnaes E., Friis-Hansen L., Norrild B., Therkildsen M.H., Glud M., Rossing M., Lajer H., Svane D., Skotte L. (2012). The role of mirnas in human papilloma virus (hpv)-associated cancers: Bridging between hpv-related head and neck cancer and cervical cancer. Br. J. Cancer.

[11] Smeets S.J., Hesselink A.T., Speel E.J., Haesevoets A., Snijders P.J., Pawlita M., Meijer C.J., Braakhuis B.J., Leemans C.R., Brakenhoff R.H. (2007). A novel algorithm for reliable detection of human papillomavirus in paraffin embedded head and neck cancer specimen. Int. J. Cancer.

[12] Nasman A., Nordfors C., Grun N., Munck-Wikland E., Ramqvist T., Marklund L., Lindquist D., Dalianis T. (2013). Absent/weak cd44 intensity and positive human papillomavirus (hpv) status in oropharyngeal squamous cell carcinoma indicates a very high survival. Cancer Med..

[13] Nasman A., Bersani C., Lindquist D., Du J., Ramqvist T., Dalianis T. (2017). Human papillomavirus and potentially relevant biomarkers in tonsillar and base of tongue squamous cell carcinoma. Anticancer Res..

[14] Badoual C., Hans S., Rodriguez J., Peyrard S., Klein C., Agueznay Nel H., Mosseri V., Laccourreye O., Bruneval P., Fridman W.H. (2006). Prognostic value of tumor-infiltrating cd4+ t-cell subpopulations in head and neck cancers. Clin. Cancer Res..

[15] Nasman A., Andersson E., Marklund L., Tertipis N., Hammarstedt-Nordenvall L., Attner P., Nyberg T., Masucci G.V., Munck-Wikland E., Ramqvist T. (2013). Hla class i and ii expression in oropharyngeal squamous cell carcinoma in relation to tumor hpv status and clinical outcome. PLoS ONE.

[16] Nasman A., Andersson E., Nordfors C., Grun N., Johansson H., Munck-Wikland E., Massucci G., Dalianis T., Ramqvist T. (2013). Mhc class i expression in hpv positive and negative tonsillar squamous cell carcinoma in correlation to clinical outcome. Int. J. Cancer.

[17] Tertipis N., Haeggblom L., Grun N., Nordfors C., Nasman A., Dalianis T., Ramqvist T. (2015). Reduced expression of the antigen processing machinery components tap2, lmp2, and lmp7 in tonsillar and base of tongue cancer and implications for clinical outcome. Transl. Oncol..

[18] Tertipis N., Haeggblom L., Nordfors C., Grun N., Nasman A., Vlastos A., Dalianis T., Ramqvist T. (2014). Correlation of lmp10 expression and clinical outcome in human papillomavirus (hpv) positive and hpv-negative tonsillar and base of tongue cancer. PLoS ONE.

[19] Oguejiofor K., Galletta-Williams H., Dovedi S.J., Roberts D.L., Stern P.L., West C.M. (2017). Distinct patterns of infiltrating cd8+ t cells in hpv+ and cd68 macrophages in hpv-oropharyngeal squamous cell carcinomas are associated with better clinical outcome but pd-l1 expression is not prognostic. Oncotarget.

[20] Assarsson E., Lundberg M., Holmquist G., Bjorkesten J., Thorsen S.B., Ekman D., Eriksson A., Rennel Dickens E., Ohlsson S., Edfeldt G. (2014). Homogenous 96-plex pea immunoassay exhibiting high sensitivity, specificity, and excellent scalability. PLoS ONE.

[21] Nasman A., Romanitan M., Nordfors C., Grun N., Johansson H., Hammarstedt L., Marklund L., Munck-Wikland E., Dalianis T., Ramqvist T. (2012). Tumor infiltrating cd8^+^ and foxp3^+^ lymphocytes correlate to clinical outcome and human papillomavirus (hpv) status in tonsillar cancer. PLoS ONE.

[22] Nordfors C., Grun N., Tertipis N., Ahrlund-Richter A., Haeggblom L., Sivars L., Du J., Nyberg T., Marklund L., Munck-Wikland E. (2013). Cd8^+^ and cd4^+^ tumour infiltrating lymphocytes in relation to human papillomavirus status and clinical outcome in tonsillar and base of tongue squamous cell carcinoma. Eur. J. Cancer.

[23] Partlova S., Boucek J., Kloudova K., Lukesova E., Zabrodsky M., Grega M., Fucikova J., Truxova I., Tachezy R., Spisek R. (2015). Distinct patterns of intratumoral immune cell infiltrates in patients with hpv-associated compared to non-virally induced head and neck squamous cell carcinoma. Oncoimmunology.

[24] Messina J.L., Fenstermacher D.A., Eschrich S., Qu X., Berglund A.E., Lloyd M.C., Schell M.J., Sondak V.K., Weber J.S., Mule J.J. (2012). 12-chemokine gene signature identifies lymph nod × 10−like structures in melanoma: Potential for patient selection for immunotherapy?. Sci. Rep..

[25] Sher Y.P., Chou C.C., Chou R.H., Wu H.M., Wayne Chang W.S., Chen C.H., Yang P.C., Wu C.W., Yu C.L., Peck K. (2006). Human kallikrein 8 protease confers a favorable clinical outcome in non-small cell lung cancer by suppressing tumor cell invasiveness. Cancer Res..

[26] Ma W., Gilligan B.M., Yuan J., Li T. (2016). Current status and perspectives in translational biomarker research for pd-1/pd-l1 immune checkpoint blockade therapy. J. Hematol. Oncol..

[27] Honeychurch J., Cheadle E.J., Dovedi S.J., Illidge T.M. (2015). Immuno-regulatory antibodies for the treatment of cancer. Expert Opin. Biol. Ther..

[28] Hong A.M., Vilain R.E., Romanes S., Yang J., Smith E., Jones D., Scolyer R.A., Lee C.S., Zhang M., Rose B. (2016). Pd-l1 expression in tonsillar cancer is associated with human papillomavirus positivity and improved survival: Implications for anti-pd1 clinical trials. Oncotarget.

[29] Felder M., Kapur A., Gonzalez-Bosquet J., Horibata S., Heintz J., Albrecht R., Fass L., Kaur J., Hu K., Shojaei H. (2014). Muc16 (ca125): Tumor biomarker to cancer therapy, a work in progress. Mol. Cancer.

[30] Slebos R.J., Jehmlich N., Brown B., Yin Z., Chung C.H., Yarbrough W.G., Liebler D.C. (2013). Proteomic analysis of oropharyngeal carcinomas reveals novel hpv-associated biological pathways. Int. J. Cancer.

[31] Sewell A., Brown B., Biktasova A., Mills G.B., Lu Y., Tyson D.R., Issaeva N., Yarbrough W.G. (2014). Revers × 10−phase protein array profiling of oropharyngeal cancer and significance of pik3ca mutations in hpv-associated head and neck cancer. Clin. Cancer Res..

[32] Pazina T., Shemesh A., Brusilovsky M., Porgador A., Campbell K.S. (2017). Regulation of the functions of natural cytotoxicity receptors by interactions with diverse ligands and alterations in splice variant expression. Front. Immunol..

[33] Freud A.G., Mundy-Bosse B.L., Yu J., Caligiuri M.A. (2017). The broad spectrum of human natural killer cell diversity. Immunity.

[34] Mirghani H., Ugolin N., Ory C., Lefevre M., Baulande S., Hofman P., St Guily J.L., Chevillard S., Lacave R. (2014). A predictive transcriptomic signature of oropharyngeal cancer according to hpv16 status exclusively. Oral Oncol..

[35] Gaykalova D.A., Manola J.B., Ozawa H., Zizkova V., Morton K., Bishop J.A., Sharma R., Zhang C., Michailidi C., Considine M. (2015). Nf-kappab and stat3 transcription factor signatures differentiate hpv-positive and hpv-negative head and neck squamous cell carcinoma. Int. J. Cancer.

[36] Keck M.K., Zuo Z., Khattri A., Stricker T.P., Brown C.D., Imanguli M., Rieke D., Endhardt K., Fang P., Bragelmann J. (2015). Integrative analysis of head and neck cancer identifies two biologically distinct hpv and three non-hpv subtypes. Clin. Cancer Res..

[37] Wilkie M.D., Emmett M.S., Santosh S., Lightbody K.A., Lane S., Goodyear P.W., Sheard J.D., Boyd M.T., Pritchard-Jones R.O., Jones T.M. (2016). Relative expression of vascular endothelial growth factor isoforms in squamous cell carcinoma of the head and neck. Head Neck.

[38] Fei J., Hong A., Dobbins T.A., Jones D., Lee C.S., Loo C., Al-Ghamdi M., Harnett G.B., Clark J., O’Brien C.J. (2009). Prognostic significance of vascular endothelial growth factor in squamous cell carcinomas of the tonsil in relation to human papillomavirus status and epidermal growth factor receptor. Ann. Surg. Oncol..

[39] Zhang L.P., Chen H.L. (2017). Increased vascular endothelial growth factor expression predicts a worse prognosis for laryngeal cancer patients: A meta-analysis. J. Laryngol. Otol..

[40] Zhao S.F., Yang X.D., Lu M.X., Sun G.W., Wang Y.X., Zhang Y.K., Pu Y.M., Tang E.Y. (2013). Prognostic significance of vegf immunohistochemical expression in oral cancer: A meta-analysis of the literature. Tumour Biol..

[41] Roudnicky F., Poyet C., Wild P., Krampitz S., Negrini F., Huggenberger R., Rogler A., Stohr R., Hartmann A., Provenzano M. (2013). Endocan is upregulated on tumor vessels in invasive bladder cancer where it mediates vegf-a-induced angiogenesis. Cancer Res..

[42] Sagara A., Igarashi K., Otsuka M., Kodama A., Yamashita M., Sugiura R., Karasawa T., Arakawa K., Narita M., Kuzumaki N. (2017). Endocan as a prognostic biomarker of tripl × 10−negative breast cancer. Breast Cancer Res. Treat..

[43] Huang X., Chen C., Wang X., Zhang J.Y., Ren B.H., Ma D.W., Xia L., Xu X.Y., Xu L. (2016). Prognostic value of endocan expression in cancers: Evidence from meta-analysis. Oncol. Targets Ther..

[44] Irshad K., Mohapatra S.K., Srivastava C., Garg H., Mishra S., Dikshit B., Sarkar C., Gupta D., Chandra P.S., Chattopadhyay P. (2015). A combined gene signature of hypoxia and notch pathway in human glioblastoma and its prognostic relevance. PLoS ONE.

[45] Tudisco L., Orlandi A., Tarallo V., De Falco S. (2017). Hypoxia activates placental growth factor expression in lymphatic endothelial cells. Oncotarget.

[46] van der Veeken J., Oliveira S., Schiffelers R.M., Storm G., van Bergen En Henegouwen P.M., Roovers R.C. (2009). Crosstalk between epidermal growth factor receptor-and insulin-like growth factor-1 receptor signaling: Implications for cancer therapy. Curr. Cancer Drug Targets.

[47] Wang Y., Yuan J.L., Zhang Y.T., Ma J.J., Xu P., Shi C.H., Zhang W., Li Y.M., Fu Q., Zhu G.F. (2013). Inhibition of both egfr and igf1r sensitized prostate cancer cells to radiation by synergistic suppression of DNA homologous recombination repair. PLoS ONE.

[48] Matsumoto F., Fujimaki M., Ohba S., Kojima M., Yokoyama J., Ikeda K. (2015). Relationship between insulin-like growth factor-1 receptor and human papillomavirus in patients with oropharyngeal cancer. Head Neck.

[49] Bersani C., Mints M., Tertipis N., Haeggblom L., Sivars L., Ahrlund-Richter A., Vlastos A., Smedberg C., Grun N., Munck-Wikland E. (2017). A model using concomitant markers for predicting outcome in human papillomavirus positive oropharyngeal cancer. Oral Oncol..

[50] Benjamini Y., Hochberg Y. (1995). Controlling the false discovery rate -a practical and powerful approach to multiple testing. J. Roy. Stat. Soc. B Met..

