# Supplementary figures and images for: Protein Expression in Tonsillar and Base of Tongue Cancer and in Relation to Human Papillomavirus (HPV) and Clinical Outcome
Source: Int J Mol Sci. 2018 Mar 25;19(4):978. doi: 10.3390/ijms19040978 (PMC5979357; doi:10.3390/ijms19040978)

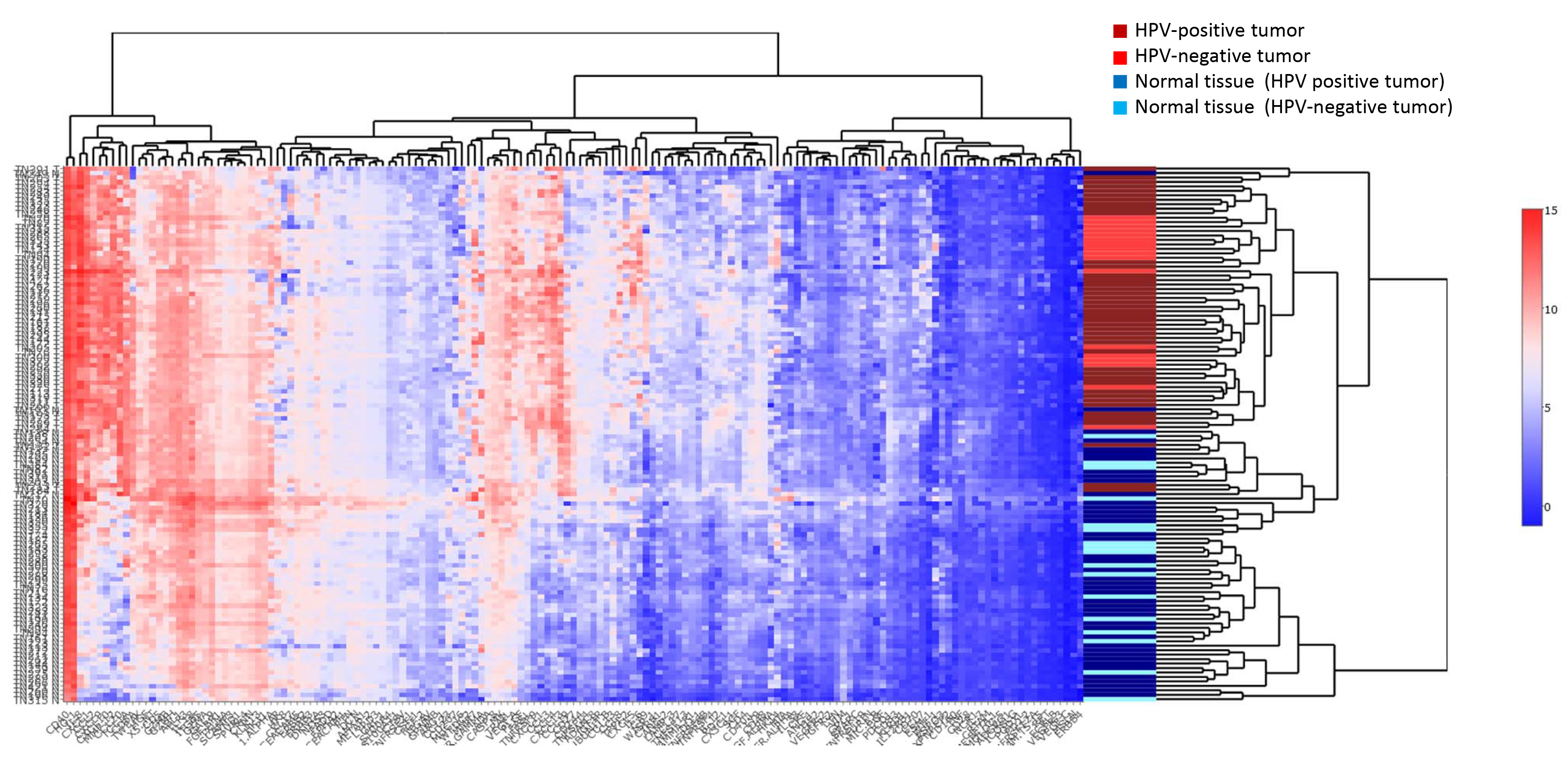

Supplement: Supplementary file 1 [file ijms-19-00978-s001.zip › Figure S1 Heatmap all included samples.tif]

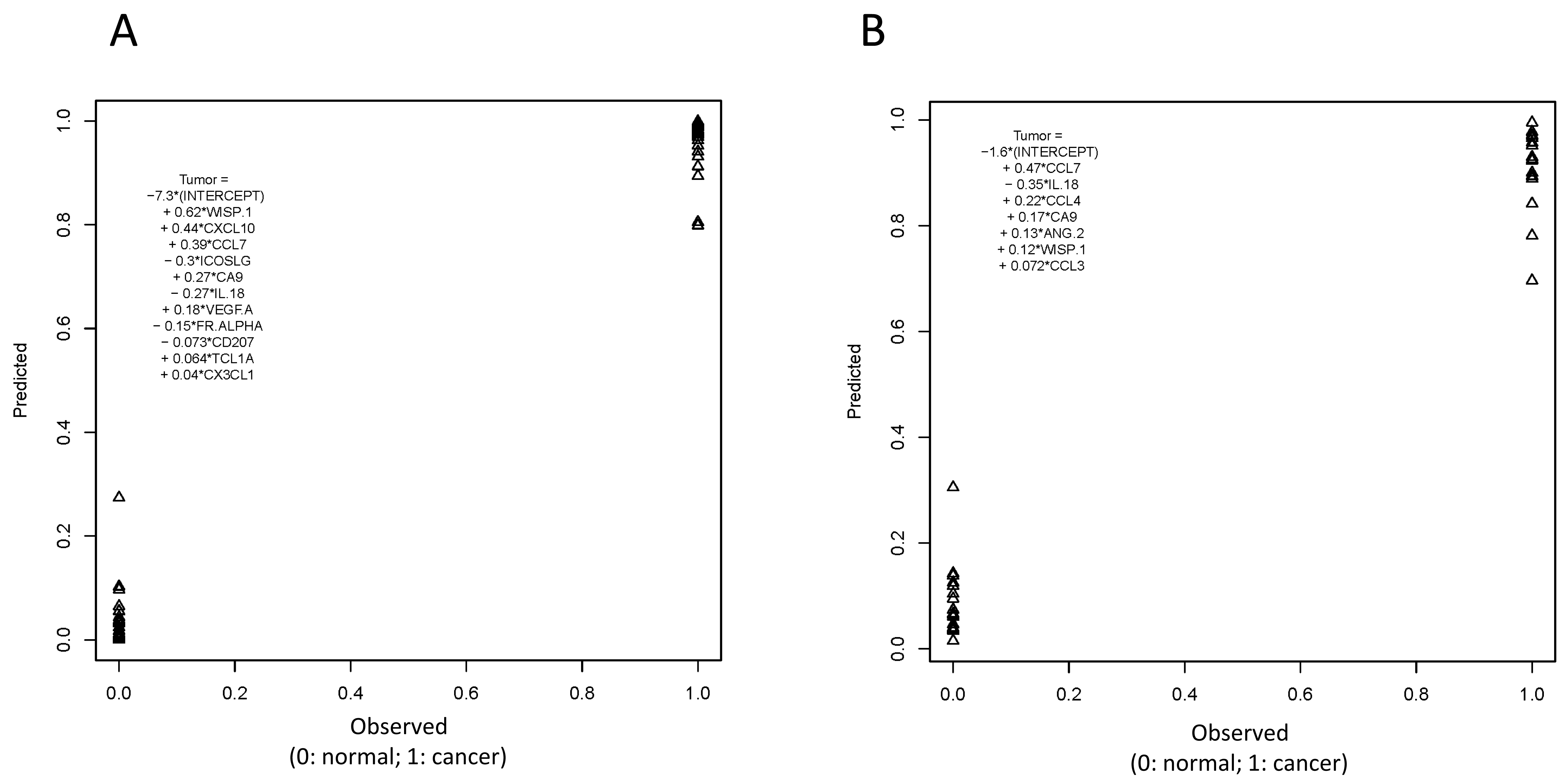

Supplement: Supplementary file 1 [file ijms-19-00978-s001.zip › Figure S2 Prediction of HPV positive or negative tumors.tif]
